# Binding of Herpes Simplex Virus Type-1 Virions Leads to the Induction of Intracellular Signalling in the Absence of Virus Entry

**DOI:** 10.1371/journal.pone.0009560

**Published:** 2010-03-05

**Authors:** Iain J. MacLeod, Tony Minson

**Affiliations:** Division of Virology, Department of Pathology, University of Cambridge, Cambridge, United Kingdom; Institute of Molecular and Cell Biology, Singapore

## Abstract

The envelope of HSV-1 contains a number of glycoproteins, four of which are essential for virus entry. Virus particles lacking gB, gD, gH or gL are entry-defective, although these viruses retain the ability to bind to the plasma membrane via the remaining glycoproteins. Soluble forms of gD have been shown to trigger the nuclear translocation of the NF-κB transcriptional complex in addition to stimulating the production of Type I interferon. By taking advantage of the entry-defective phenotype of glycoprotein-deficient HSV-1 virus particles, the results presented here show that binding of virions to cellular receptors on the plasma membrane is sufficient to stimulate a change in cellular gene expression. Preliminary microarray studies, validated by quantitative real-time PCR, identified the differential expression of cellular genes associated with the NF-κB, PI3K/Akt, Jak/Stat and related Jak/Src pathways by virions lacking gB or gH but not gD. Gene induction occurred at a few particles per cell, corresponding to physiological conditions during primary infection. Reporter assay studies determined that NF-κB transcriptional activity is stimulated within an hour of HSV-1 binding, peaks between two and three hours post-binding and declines to background levels by five hours after induction. The immediate, transient nature of these signalling events suggests that HSV-1 glycoproteins, particularly gD, may alter the cellular environment pre-entry so as to condition the cell for viral replication.

## Introduction

Subjugation of the intracellular environment by viruses is essential to ensure the effective expression and replication of the viral genome to allow production of progeny virions. One such viral strategy involves hijacking signalling pathways that ultimately control host gene transcription. Interactions between intracellular viral proteins and cellular kinases responsible for signal transduction are a means by which to achieve this. However, there is growing evidence to support the premise that glycoproteins on the surface of virus particles may trigger intracellular signalling pathways by interacting with their cognate receptors on the host cell membrane [1].

Binding of HSV-1 to permissive cells occurs through viral glycoproteins on the viral envelope interacting with specific receptors on the cell surface, triggering fusion of the plasma membrane with the outer envelope. Five of these glycoproteins are known to be involved in virion binding to the cell surface: gB, gC, gD and the heterodimer gH-L. Of the five, only gC is dispensable for allowing productive infection as deletion of gB, gD or gH-L results in an entry-defective phenotype [2] [3]. gD is known to bind independently to HvEM, nectin-1 and nectin-2, whereas gH interacts with the αvβ3 integrin, with the paired immunoglobulin-like receptor, PILRα, acting as a receptor for gB [2] [4] [5].

One of the first studies to examine whether HSV-1 glycoproteins play a role in the induction of signalling found that gD was able to block Fas-mediated apoptosis [6]. U937 monocytoid cells were rendered resistant to apoptosis after infection with UV-inactivated HSV virions, when co-cultured with a stably transfected HSV-1 gD-expressing cell line or after treatment with soluble gD. Inhibition of NF-KB signalling by the introduction of a dominant-negative NF-κB repressor abolished protection from gD-induced Fas-mediated apoptosis. More specifically, the interaction of gD with one of the gD receptors, HVEM, was involved in preventing apoptosis induction [1].

There are reports showing that HSV-1 triggered the translocation of NF-κB by six hours post-infection [7]. However, it has come to light that HSV-1 may induce two distinct phases of NF-κB activity. The initial phase is transient, lasting only two hours, and occurred shortly after viral adsorption by both wild-type and UV-inactivated virus particles. This correlates with evidence showing that supernatant taken from gD-expressing cells may also cause an increase in NF-KB binding activity in monocytoid cells by 30 minutes post-treatment [6]. A second phase relied on *de novo* viral protein synthesis as it was stimulated only by replication-competent HSV-1 and not by UV-inactivated virions [8]. During this second phase, NF-κB complexes were shown to have associated with their consensus sequence found in the promoter region that drives ICP0 expression [8].

In comparison to wild-type gD, a mutated form of gD that was unable to bind to HVEM did not stimulate NF-KB activity in co-cultured monocytoid cells [9]. HEp-2 cells, which do not express HVEM, lacked detectable NF-κB signalling after infection with UV-inactivated HSV-1, implying that gD-mediated NF-κB stimulation may be dependent on expression of HVEM [9].

Soluble glycoproteins from beta- and gammaherpesviruses can also stimulate intracellular signalling pathways; gB of CMV has been shown to stimulate the differential expression of cellular transcription factors and interferon-stimulated genes (ISGs), and soluble gp350 of EBV can trigger NF-κB activation [10] [11] [12].

The treatment of cells with soluble glycoproteins or UV-inactivated virus has provided strong evidence that HSV-1 binding to the plasma membrane triggers signal transduction in the host cell as a prelude to infection, but interpretation of these experiments is not straightforward. The use of soluble glycoproteins does not mimic the binding of individual virus particles and the physiological significance of the results is difficult to assess. The use of UV-inactivated particles in signalling studies simulates infection but these particles are able to enter cells and deliver virion proteins to the cytoplasm. Experiments with UV-inactivated particles do not therefore distinguish between events arising from virus binding from those resulting from virus entry.

The main focus in these studies was to examine signalling events using virus particles that are capable of binding to the cell surface but are incapable of entry due to the absence of one of the three essential glycoproteins, gB, gD or gH [13] [14] [15] [16], and to find whether signalling occurs at particle concentrations that would mirror physiological conditions.

## Materials and Methods

### Viruses and Cells

All cells other than Human Foreskin Fibroblasts (HFF) (ATCC cell line CRL-2522) were grown in Glasgow Modified Eagles Medium supplemented with 10% foetal bovine serum, 4 mM glutamine, 100 units/ml penicillin and 100 ug/ml streptomycin. HFF cells were grown in Dulbecco modified Eagles medium supplemented as described above but with the addition of 1x MEM non-essential amino acids.

Working stocks of HSV-1 SC16 were grown on HaCaT cells and assayed on Vero cells [17]. Mutant viruses lacking functional genes for gH (SCgHZ) [14], gB (HFEMdUL27-lacZ) [18] or gD (SC16gDdelZ) [18] were grown and assayed on helper cell lines expressing the corresponding glycoprotein: CR1 cells, expressing gH [19]; D6 cells expressing gB [13]; and VD60 cells expressing gD [18]. All stocks were grown using an MOI of 0.1.

Purified preparations of WT virus, or of virions lacking individual glycoproteins, were produced as previously described [20]. Purified preparations were assayed for particle numbers using electron microscopy [21] and for infectivity by plaque assay on Vero cells or, in the case of glycoprotein-deficient virions, on the relevant helper cell line. Preparations of WT virions had a particle to infectivity ratio of 3×10^1^. Preparations of gH-negative, gD-negative and gB negative virions had particle to infectivity ratios of 2×10^10^, 5×10^6^ and 3×10^6^ respectively. The maximum virion dose of entry-incompetent virions used in the experiments described in this paper was 1000 particles per cell and it therefore follows that in the maximum infectivity of a preparation of an entry-defective virus (i.e. using gB-negative virions) would be less than one cell per thousand. Virus preparations were deglycosylated with 500 U of PNGase F for 18 hrs at 37°C in the absence of denaturing buffer [22]. Removal of N-linked sugars was confirmed by a shift in the electrophoretic mobility of gD on a protein denaturing gel.

### Signalling Experiments

Human foreskin fibroblast cells (HFF) were seeded at 1.5×10^6^ in a 175^2^ cm flask and grown to 90% confluence then growth-arrested in medium supplemented with only 100 U/ml Penicillin-G and 100 µg/ml Streptomycin for five days. Real-time PCR experiments were conducted with growth-arrested HFFs that were inoculated in triplicate with 1000 particles/cell of entry-defective HSV-1 in warm, serum-free medium, with parallel mock-infected cells that received an equivalent volume of PBS in warm serum-free medium. Inoculations took place at 37°C. At the indicated times after the addition of virus, the medium was removed, the cells were lysed with TRI Reagent (Sigma) and total RNA was immediately purified. For each entry-defective HSV-1 mutant, three independent biological replicates were carried out. Microarray experiments were conducted under the same conditions using wild-type HSV-1 (SC16) at an MOI of 20.

### RNA Purification and cDNA Preparation

Total RNA was isolated from TRI reagent lysates as per the manufacturer's instructions. Contaminating genomic DNA was digested with 2 U DNase I (Invitrogen) and RNA was re-purified with TRI reagent. cDNA was derived from 30 µg total RNA using anchored oligo (dT)20 primers (Invitrogen) as described in the manufacturer's instructions. For each entry-defective HSV-1 mutant, the cDNA from three independent infections were used in technical triplicates for real-time PCR reactions.

### Signalling-Specific Microarray

Microarray analysis was carried out using the Signal Transduction PathwayFinder cDNA array as per the manufacturer's instructions (Superarray). Nylon cDNA microarrays membranes were pre-hybridised with supplied sheared salmon sperm DNA at 60°C for one to two hours. The pre-hybridisation solution was replaced with the biotin-dUTP labelled cDNA probe, which was diluted in sheared salmon sperm DNA. The solution was left to hybridise at 60°C overnight. The following morning the cDNA probe was discarded and the membrane was washed twice then blocked in Solution Q, as provided by the manufacturer. After 40 minutes, the blocking solution was discarded and the membrane was incubated with a binding buffer, Solution F. After washing, the chemiluminescent substrate was incubated with the membrane for two to five minutes at room temperature. The microarray membrane was then exposed to X-ray film. Analysis was performed using GEArray Expression Analysis Suite as provided by the manufacturer (Superarray). All the data sets used in the microarray analysis are available in Table S1.

### Real-Time PCR

cDNA derived from cells that were mock-infected or inoculated with 1000 particles/cell of entry-defective ΔgB, ΔgD and ΔgH virions was used for real-time PCR analysis. Primers were designed, using the Primer3 software [23], to anneal at 60°C, with a minimum product melting temperature of 80°C (Table S2). Reactions were set-up with cDNA corresponding to 100 ng total RNA, primers (70 nM), and 10 µl SYBR Green PCR master mix (Abgene) made to a total volume of 20 µl with nuclease-free dH_2_O. A Corbett Rotorgene 3000 was used to determine the levels of SYBR green fluorescence over 40 cycles. Samples were denatured for 10 minutes at 95°C, annealed at 60°C for 30 seconds, with extension of the primer product at 72°C for 40 seconds then a final 80°C step for 20 seconds that removes background fluorescence from primer-dimers. A melt curve analysis was produced at the end of the cycling to ensure the specificity of PCR product amplification and associated SYBR green fluorescence. Relative gene expression levels between mock-inoculated and inoculated cells were calculated using the Pfaffl method with potential variations in cDNA quantity between samples normalised to the transcript for ribosomal protein L13a (RPL13a) [24]. Serial dilutions of PCR product across 8 logs were used to determine the efficiency of PCR amplification for each primer set under the above conditions so that the relative quantitation could be adjusted as defined by the Pfaffl method.

A relative change in expression of two-fold was set as a threshold for determining whether differential expression of a gene had occurred. The *p* values associated with the fold-change in expression were calculated using a Students t-test on the real-time PCR C_T_ values comparing triplicate mock-inoculated and HSV inoculated cells.

### Luciferase Assays

An NF-κB reporter assay system was designed using a pGL4-NFκB construct (Promega). Quadruplicate repeats of the NF-κB binding consensus sequence 5′-GGGAATTTCC-3′ were excised from a p-NF-κB-Luc (Gift from Dr. Heike Laman, University of Cambridge) vector using KpnI and HindIII and ligated into the pGL4.20 vector multiple cloning site using the same restriction sites. pGL4.20 was chosen as it is optimized for more efficient expression in mammalian cells, compared to previous generations of luciferase constructs, and contains fewer transcription factor consensus sequences such that background luciferase expression is reduced. HFFs were electroporated in batches of 6×10^5^ cells per cuvet (Amaxa) with 2 µg of pGL4-NF-κB, pooled together and aliquoted at 10^5^ cells per well of a 24-well plate. After 48-hours recovery in supplemented media, the transfected HFFs were serum-starved for five days. HFFs were inoculated with 1000 particles/cell of ΔgH HSV-1 and were lysed at the indicated time points in 100 µl passive lysis buffer (Promega) and 20 µl of lysate were examined for luciferase activity using 50 µl assay buffer (Promega).

### Western Blotting

gD and the major tegument protein, VP16, were detected by immunoblotting using the monoclonal antibodies LP2 and LP1, respectively [25]–[26]. Sodium dodecyl sulfate-polyacrylamide gel electrophoresis (SDS-PAGE) and immunoblotting were performed as described previously [27].

## Results

### Differential Cellular Gene Expression at Early Time-Points after Wild-Type HSV-1 Infection

Signalling-specific microarrays were chosen as an initial route to identifying signalling pathways that may be stimulated early in HSV-1 infection, and more precisely, by virion binding. The Signal Transduction PathwayFinder cDNA array from Superarray incorporates 96 targets covering 19 associated signalling pathways, in addition to two negative controls corresponding to pUC18 plasmid DNA and a ‘blank’ spot, as well as four positive controls representing the housekeeping genes for GAPDH, beta actin, cyclophilin A and ribosomal protein L13a.

Serum-starved HFFs were inoculated with 1000 particles/cell of ΔgB, ΔgD or ΔgH entry-defective HSV-1 virions. Total RNA was isolated immediately after inoculation, corresponding to 0 hpi, and 6 hpi. Changes in gene expression associated with the activation of signalling pathways were identified by comparison to the transcript abundance at 0 hpi. The results from duplicate experiments indicated that downstream gene targets of a number of signalling pathways are differentially expressed above the two-fold threshold after virion binding (Table 1). These preliminary results were then used as the basis of quantitative PCR studies.

**Table 1:** Fold change in gene expression after inoculation with entry-defective HSV-1.

Gene	Description	ΔgB	ΔgD	ΔgH
**NF-KB**				
BCL2A1	hematopoietic BCL2-related protein A1	−3.85 *	5.56	1.42
BIRC1	baculoviral IAP repeat-containing 1	−2.33	−1.12	−3.13 **
BIRC2	baculoviral IAP repeat-containing 2	3.85	3.98	1.72
BIRC3	baculoviral IAP repeat-containing 3	2.88	1.00	7.71 ***
CCL2	macrophage chemoattractant protein-1	2.32 *	1.34	2.13 *
LTA	lymphotoxin alpha (TNF superfamily, member 1)	−1.22	2.31	−1.20
NFKB1	nuclear factor of kappa light polypeptide gene enhancer in B-cells 1 (p105)	2.98 *	−1.92	3.09 **
NFKBIA	nuclear factor of kappa light polypeptide gene enhancer in B-cells inhibitor, alpha	−1.19	1.23	2.54 **
PECAM1	platelet/endothelial cell adhesion molecule (CD31 antigen)	4.60 *	1.00	3.09 *
**JAK/STAT**				
A2M	alpha-2-macroglobulin	5.07 *	1.79	2.23
CSN2	casein beta	5.23 **	−1.54	1.48
CXCL9	chemokine (C-X-C motif) ligand 9	2.22	−1.59	1.06
IRF1	interferon regulatory factor-1	1.90	−2.94	1.00
MMP10	matrix metallopeptidase 10	1.09	−1.12	2.50
NOS2A	nitric oxide synthase 2A	1.26	1.21	8.01 **
**PI3K/AKT**				
BCL2	B-cell CLL/lymphoma 2	3.54	−1.54	1.86 **
CCND1	cyclin D1	1.54	−9.09 ***	3.22 *
FN1	fibronectin 1	1.50	1.03	6.28 **
MMP7	matrix metallopeptidase 7	2.05	1.91	3.05 **
MYC	v-MYC myelocytomatosis viral oncogene homolog	2.83	1.09	4.55
WISP2	WNT1 inducible signaling pathway protein 2	−7.69 **	1.50	−4.55 ***
**Jak/Src**				
BCL2	B-cell CLL/lymphoma 2	3.54	−1.54	1.86 **
BCL2L1	BCL2-like 1; Bcl-X	−4.92	−5.00 **	−3.33
**p53**				
GADD45A	growth arrest and DNA-damage-inducible, alpha	5.22 *	−1.15	1.96
IGFBP3	insulin-like growth factor binding protein 3	−6.67 *	−1.04	1.18 *
MDM2	Mdm2 p53 binding protein homolog (mouse)	6.83 *	−1.82	2.89
**Phospholipase C**				
EGR1	early growth response 1	−2.70	−2.50 *	1.09
FAS	fas (TNF receptor superfamily, member 6)	−2.38	1.15	−2.63 *
FOS	FBJ murine osteosarcoma viral oncogene homolog	−2.78 *	1.56	−1.59
JUNB	jun B proto-oncogene	−1.01	−2.56	−1.33
PTGS2	rostaglandin-endoperoxide synthase 2	1.26	1.21	8.01
**TGF-β**				
CDKN1A	cyclin-dependent kinase inhibitor 1A	1.12 *	4.01 *	1.10
CDKN2A	cyclin-dependent kinase inhibitor 2A	1.89	1.35	−4.17 **
CDKN2B	cyclin-dependent kinase inhibitor 2B	−1.19	2.72	−2.27
CDKN2D	cyclin-dependent kinase inhibitor 2D	1.31	−4.55 **	1.05
**Retinoic Acid**				
CDX1	caudal type homeobox 1	1.31	2.05	1.09
CTSD	cathepsin D	−1.05	−3.70	−1.12
EN1	engrailed homeobox 1	−1.10	−2.22	1.00
RBP1	retinol binding protein 1, cellular	1.68	−3.03 *	1.00
**Androgen**				
CDK2	cyclin-dependent kinase 2	3.86	−1.45	3.44 **
KLK2	kallikrein-related peptidase 2	6.83 ***	−1.82	2.89
TMEPA1	prostate transmembrane protein, androgen induced 1	3.69	1.41	−3.13 ***
**Hedgehog**				
BMP4	bone morphogenetic protein 4	−2.22 *	−2.38	−1.67

HFFs were stimulated with 1000 particles/cell of ΔgB, ΔgD or ΔgH HSV-1 for six hours and a cDNA microarray corresponding to targets of 19 signalling pathways was used to detect changes in cellular gene expression when compared to mock-infected. Infections and mock infections were carried out in duplicate. A number of signalling pathways were shown to be stimulated by one or more of the entry-defective mutants. These data were used to independently verify changes in expression using real-time PCR.

Using the same methodology, serum-starved HFF cells were exposed to 1000 particles per cell of virions lacking either gD, gB or gH and RNA was extracted for cDNA synthesis immediately (0 h) or after 6 h. Table 2 gives the results of three independent experiments for those genes that were differentially expressed between 0 h and 6 h post treatment and is compared to the initial microarray results in Table 3. It is striking that in virtually every instance where gene expression is modified by virion binding, gD appears to be the key effector. Virions lacking gH or gB had were capable of altering cellular gene expression while those lacking gD had no significant effect. The results can be summarised as follows.

**Table 2:** Independent confirmation of microarray data by real-time PCR.

Gene	Description	ΔgB	ΔgD	ΔgH
**NF-KB**				
BIRC2	baculoviral IAP repeat-containing 2	3.66 *	1.20	2.92
BIRC3	baculoviral IAP repeat-containing 3	4.45 **	1.27	3.71 **
CCL2	macrophage chemoattractant protein-1	2.09	−1.51	3.16 **
NFKB1	nuclear factor of kappa light polypeptide gene enhancer in B-cells 1 (p105)	3.54 ***	1.13	2.61 *
NFKBIA	nuclear factor of kappa light polypeptide gene enhancer in B-cells inhibitor, alpha	2.35 ***	1.07	1.48 *
NFKBIB	nuclear factor of kappa light polypeptide gene enhancer in B-cells inhibitor, beta	2.72 **	1.16	1.40
PECAM1	platelet/endothelial cell adhesion molecule (CD31 antigen)	3.49 **	1.22	4.44 **
REL	v-REL reticuloendotheliosis viral oncogene homolog (C-REL)	2.05	−1.25	2.63 *
**JAK/STAT**				
A2M	alpha-2-macroglobulin	3.54 **	1.77	2.55 **
MMP10	matrix metallopeptidase 10	2.87 **	1.52	2.62 ***
NOS2A	nitric oxide synthase 2A	5.00 **	−1.09	4.41 *
**PI3K/AKT**				
BCL2	B-cell CLL/lymphoma 2	2.86 *	−2.33 *	2.13
MMP7	matrix metallopeptidase 7	3.67 *	−1.03	2.04
MYC	v-MYC myelocytomatosis viral oncogene homolog	4.53 ***	−1.04	2.67 *
**Jak/Src**				
BCL2	B-cell CLL/lymphoma 2	2.86 *	−2.33 *	2.13
BCL2L1	BCL2-like 1; Bcl-X	2.56 **	−1.92 *	2.09 **
**Androgen**				
CDK2		3.01**	1.18	3.08**

Note.

*p<0.05.

**p≤0.01.

***p≤0.001.

HFFs were stimulated with 1000 particles/cell of entry-defective HSV-1 and changes in cellular gene expression were detected by real-time PCR at 6 hours post-inoculation. NF-κB responsive genes appear to be up-regulated by binding of either ΔgB or ΔgH virions to the cell. The increase in NF-κB-associated transcripts appears specific as not all targets were differentially regulated. A similar pattern of gene expression is seen for Jak/Stat pathway targets in that ΔgD virions appear to be unable to stimulate signalling whereas ΔgB and ΔgH binding can. MMP7 is only associated with the PI3 Kinase/Akt pathway and coupled with the up-regulation of BCL2, CCND1 and MYC, it would appear that binding may be sufficient to also stimulate this pathway, although not by virions lacking in gD. The Jak/Src pathway only contains two targets on the signalling specific microarray, one of which, BCL2, was associated with other pathways.

**Table 3:** Comparison of microarray with real-time PCR data for differentially expressed genes.

Gene	ΔgB		ΔgD		ΔgH	
	Microarray	Real-Time	Microarray	Real-Time	Microarray	Real-Time
**NF-KB**						
BIRC2	3.66	3.85	1.20	3.98	2.92	−1.01
BIRC3	4.45	2.88	1.27	1.00	3.71	7.71
CCL2	2.09	2.32	−1.51	1.34	3.16	2.13
NFKB1	3.54	2.98	1.13	−1.92	2.61	3.09
NFKBIA	2.35	−1.19	1.07	1.23	1.48	2.54
NFKBIB	2.72	-	1.16	-	1.40	-
PECAM1	3.49	4.60	1.22	1.00	4.44	3.09
REL	2.05	-	−1.25	-	2.63	-
**JAK/STAT**						
A2M	3.54	5.07	1.77	1.79	2.55	2.23
MMP10	2.87	1.09	1.52	−1.12	2.62	2.50
NOS2A	5.00	1.26	−1.09	1.21	4.41	8.01
**PI3K/AKT**						
BCL2	2.86	1.86	−2.33	−1.54	2.13	1.86
MMP7	3.67	2.05	−1.03	1.91	2.04	3.05
MYC	4.53	2.83	−1.04	2.85	2.67	2.48
**Jak/Src**						
BCL2	2.86	1.86	−2.33	−1.54	2.13	1.86
BCL2L1	2.56	1.42	−1.92	−5.00	2.09	−1.18
**Androgen**						
CDK2	3.01	3.86	1.18	−1.45	3.08	3.44

There was a degree of correlation between the change in expression determined by microarray studies and those confirmed by real-time PCR, particularly for genes under the control of NF-κB. Note: *rel* and *nfkbib* were not present on the original signalling-specific microarray, but were included in the real-time analysis, as they are known to be up-regulated as part of the NF-κB feedback mechanism.

### JAK/STAT and JAK/Src Pathways

The JAK/STAT and related JAK/Src pathway show a similar response to that seen for NF-κB. Of the JAK/STAT-responsive genes, only NOS2A is regulated by more than one pathway, with ΔgB and ΔgH virions inducing their transcription and ΔgD particles having no significant effect (Table 2). Although the JAK/Src pathway has only two targets on the signalling microarrays - upon which the real-time PCR experiments were based - BCL2L1 is unique to the pathway, with BCL2 expression regulated by multiple signalling routes. Interestingly, while ΔgB and ΔgH virions up-regulated BCL2 and BCL2L1, ΔgD virus particles appeared to down-regulate both targets by a significant amount.

### PI3K/Akt Pathway

Induction of this pathway may be responsible for the upregulation of CCND1, MYC, BCL2 and MMP7 after inoculation with ΔgB and ΔgH but not ΔgD HSV-1 (Table 2). Differential expression of CCND1 and MYC can be regulated by the Wnt and PI3K signalling pathways, with MYC also being controlled by Protein Kinase C. The up-regulation of MMP7, which is the only target unique to the PI3K pathway that is differentially expressed, highlights the potential crosstalk between signalling pathways and cannot exclude the involvement of PI3 kinase signalling as a pathway leading to transcription of all four cellular genes.

### NF-κB Pathway

Both ΔgB and ΔgH virions are capable of stimulating an NF-kB response (Table 2), a result that correlates with the preliminary microarray data (Table 1). Transcripts for both BIRC-2 and BIRC-3 are increased above the two-fold threshold after inoculation with ΔgB and ΔgH HSV-1 but not ΔgD. There appears to be no change in BIRC-1 expression after inoculation with any of the three entry-defective mutants. Components of the NF-κB transcriptional complex, NFKB1 and REL, showed similar levels of increased transcription after binding by ΔgB and ΔgH virions, a response that was absent in cells stimulated with gD-deficient virions. Two markers of inflammation, CCL2 and PECAM1, also showed a significant change in gene expression at 6 hpi with entry-defective HSV-1 lacking gB or gH but not gD.

The downstream signalling appears specific as not all NF-κB targets were up-regulated. The NF-κB repressors, NFKBIA and NFKBIB, were only up-regulated by ΔgB virion binding (Table 2).

### Kinetics of NF-κB Activation

**Figure 1:**
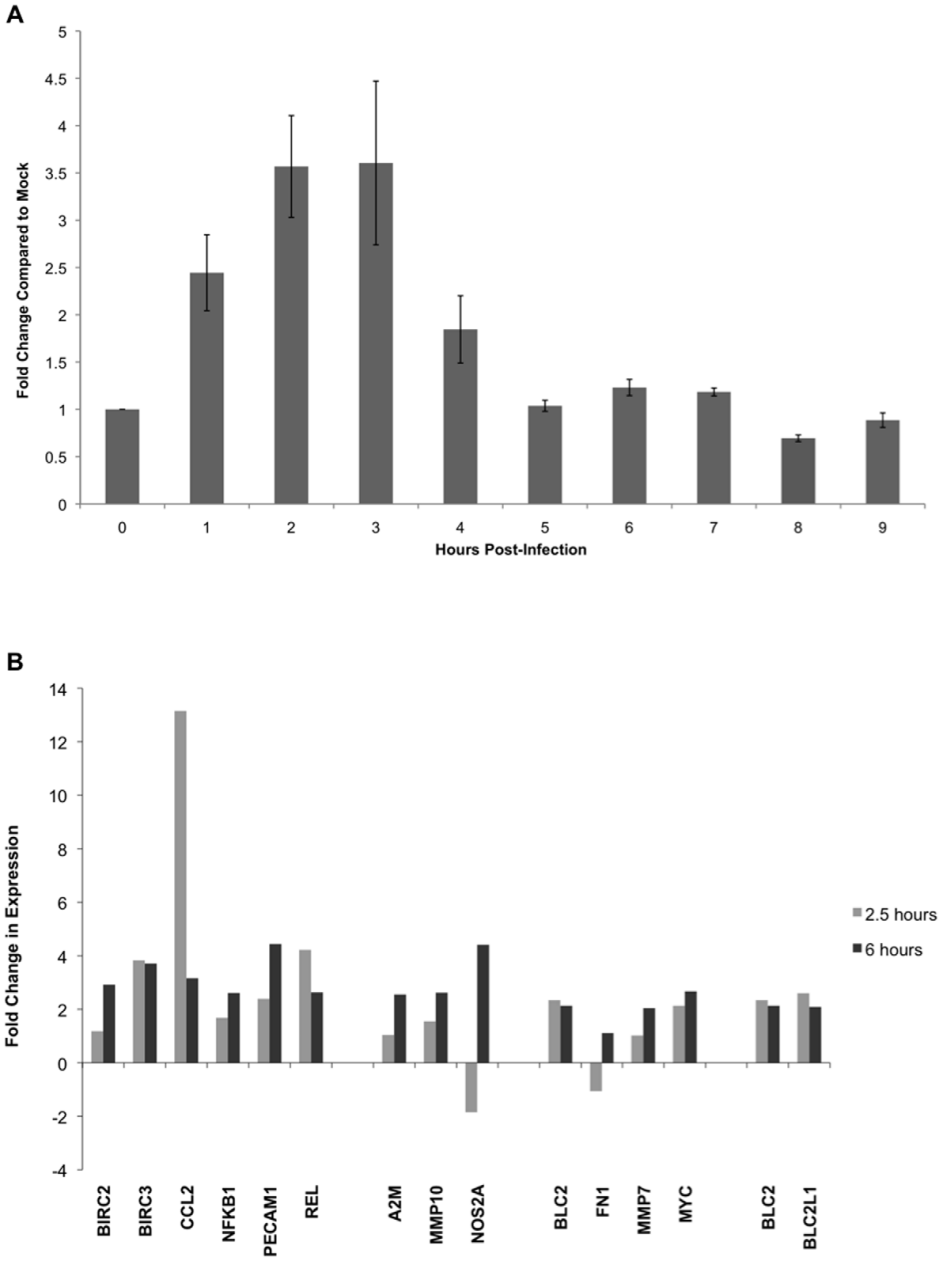
Kinetics of signalling activation. (A) An NF-κB luciferase construct was transfected into HFF, which were left to serum-starve for five days. Cells were then mock-infected or inoculated with 1000 particles/cell of entry-defective ΔgH HSV-1 then lysed at the indicated times and assayed for luciferase activity. Error bars are the +/− standard error across triplicate luciferase assays for each biological duplicate. (B) NF-κB, JAK/STAT, JAK/Src and PI3K/Akt-responsive genes that were previously shown to be differentially expressed at 6 hours post-inoculation with ΔgB and ΔgH virions were examined for changes in expression at two-and-a-half hours after inoculation with 1000 particles/cell of ΔgH virions. The induction of JAK/STAT and PI3K/Akt targets is not significantly up-regulated above the two-fold threshold by two-and-a-half hours post-inoculation. A number of NF-κB-associated transcripts are significantly up-regulated, with *ccl2* showing a far greater induction at two-and-a-half hours than six hours. Both JAK/Src targets, *bcl2* and *bcl2l1* are induced to similar levels found at six hours post-inoculation. Error bars are the +/− standard error across triplicate biological replicates.

To confirm that virion binding induces NF-κB and to examine the kinetics, an NF-κB promoter-driven luciferase construct was transfected into HFF cells and cells were treated with ΔgH virions (1000 particles per cell) or were mock-treated. Cells were harvested every hour until 9 hr post treatment, lysed and assayed for luciferase activity in triplicate. Figure 1(a) shows the fold-change in luciferase activity of those HFFs inoculated with ΔgH in comparison to mock-inoculated cells at the same time point. Biological duplicates of the inoculations were performed with the lysates used in triplicate reporter assays. At 1 hpi, there is an average of 2.4 fold change in luciferase activity from the NF-κB reporter, rising to 3.5 at 2 hpi and is maintained through to 3 hpi whereby it drops to 1.8 at 4 hpi, which correlates with the half-life of luciferase [28]. By 5 hpi, and until 9 hpi, luciferase activity drops to baseline levels at, or near, 1-fold change. Error bars represent the standard error across the six readings taken from technical triplicates on biological duplicates.

As induction of the NF-κB reporter construct occurred within one hour of inoculation with ΔgH virions and peaked at around two-and-a-half hours post-inoculation, then the transcripts previously shown in Table 2 to be up-regulated as a result of ΔgH virions binding were examined for changes in transcript levels at two-and-a-half hours after inoculation with ΔgH. Figure 1(b) compares expression levels at two-and-a-half and six hours post-inoculation. Transcripts for the NF-κB-responsive chemokine CCL2 were up-regulated to a greater degree at two-and-a-half hours than at six hours post-inoculation. Of the NF-κB-responsive genes, REL transcription was induced to a higher level whereas PECAM1 transcript abundance was reduced in comparison to six hours. Although JAK/STAT-responsive targets were not differentially expressed by two-and-a-half hours, the JAK/Src targets BCL2 and BCL2L1 were up-regulated above the two-fold arbitrary threshold to similar transcript levels that were seen at six hours post-inoculation.

### Interferogenic Capacity of Entry-Defective Virions

The initial microarray screen was uninformative with respect to interferon signalling, but this was of interest because soluble gD has been reported to induce IFN [29], while, in contrast, ISG induction is reported to require virion entry but not *de novo* protein synthesis ^[30]^. Primers were designed to Type I interferons, α (IFNA1) and ® (IFNB1), and the newly designated Type III interferon λ (IL29). Human fibroblasts do not produce interferon gamma. Additionally, the cDNA preparation was used to identify any change in the abundance of ISGs. Both ΔgB and ΔgH virions were able to stimulate the up-regulation of IFN-α expression 2.81 fold and 2.34 fold, respectively, with a high degree of statistical significance (p≤0.05) (Table 4). HSV-1 particles lacking gD were unable to do so, with a fold change of −1.11 and a p = 0.15 (Table 4).

**Table 4:** Interferon response to HSV-1 virion binding.

Name	Description	ΔgB	ΔgD	ΔgH	MOI 0.01
IFNA1	interferon, alpha 1	2.63 *	−1.11	2.25 *	−1.23
IFNB1	interferon, beta 1, fibroblast	3.01 *	2.15 *	−1.07	−1.18
IL29	interleukin 29 (interferon, lambda 1)	1.51	−1.33	−1.69	1.11
ISG54	IFN-induced protein 54	1.20	1.57	−1.54	−1.45
IRF1	interferon regulatory factor 1	−1.21	1.07	1.36	1.00
IRF3	interferon regulatory factor 3	1.14	−1.10	1.05	1.08
IRF7	interferon regulatory factor 7	1.19	1.03	−1.05	−1.23
IRF9	interferon regulatory factor 9	−1.23	1.03	1.44	−1.33

Note.

*p<0.05.

Binding by ΔgB and ΔgH virions up-regulated the expression of IFN-α whereas ΔgB and ΔgD virions were able to stimulate an increase in IFN-β. Infection with wild-type HSV-1 at an MOI of 0.01 was insufficient to cause similar increases in interferon expression. Interferon-stimulated genes were not up-regulated above the two-fold threshold by binding of any of the entry-defective HSV-1 mutants. Wild-type infection at a low MOI was also insufficient to cause in increase in ISG transcripts when compared to mock-infected cells.

IFN-® transcript levels were up-regulated 2.22 fold by ΔgB virions and 2.06 fold (p = 0.01) by ΔgD virions, again with a high degree of statistical significance. Inoculation with virus particles lacking gH led to a −1.07 fold change (p = 0.44) in IFN-® expression when compared to mock-infected cultures. All three entry-defective mutants were unable to modulate the expression of IFN-λ above the arbitrary two-fold threshold.

The glycosylation status of IFN-inducing viral glycoproteins is a potential determinant of induction efficacy [22] [31]. HSV-1 gD has three potential N-glycosylation sites but lacks O-linked glycosylation. ΔgH virions, that retain expression of gD, were treated with the endoglycosidase, PNGase F, which removes N-linked sugars. A long incubation was required as the denaturing buffer was excluded from the reaction to avoid conformational changes that might abrogate virion binding. As previously shown, wild-type virions treated in a similar manner retained infectivity (Figure S1) [22].

HFF were inoculated as above with ΔgH virions that had been incubated for 18 hrs in the presence or absence of PNGase. Glycosylated and deglycosylated ΔgH virions stimulated a 2.23 and 2.28 fold increase in IFN-α transcription, respectively. These results suggest that glycosylation has no impact on the interferogenic properties of ΔgH virions, unlike the glycoproteins of MHV and TGEV, which appear to stimulate Type I IFN through a lectin-like action, HSV-1 gD may trigger IFN up-regulation through a different mechanism, possibly analogous to that used to stimulate other signalling pathways.

Five interferon-stimulated genes were chosen to examine whether they were differentially expressed by virion binding. IFN-induced protein 54 (ISG54) transcripts are routinely used as a marker of ISG induction. Binding by ΔgB, ΔgD or ΔgH virions was insufficient to cause changes in the transcript abundance of interferon regulatory factor-1, -3, -7, -9 or ISG54 (Table 4).

To ensure that these observations could not result from a background of infection by competent virus, parallel cultures were infected with wild type virus at an MOI of 0.01, a level of infection some tenfold higher than would result from the ‘worst case’ scenario using preparations of entry defective virions (see Methods). These cultures exhibited no induction of IFN or ISGs (Table 4).

### Effects of ΔgH Virion Multiplicity on Stimulating Signalling

All the experimental work described above was performed using treatments with 1000 virus particles per cell, a condition that would be unlikely to pertain in natural primary infections. To establish whether much lower virion numbers would be effective, HFFs were serum-starved for five days then mock-inoculated or inoculated with 1, 10, 100 or 1000 particles per cell of ΔgH HSV-1 for six hours. Infections were carried out in duplicate with mock- inoculated controls that received an equivalent volume of PBS. Total RNA was purified and reverse transcribed, with the cDNA from each duplicate inoculation used in triplicate for real-time PCR.

**Figure 2:**
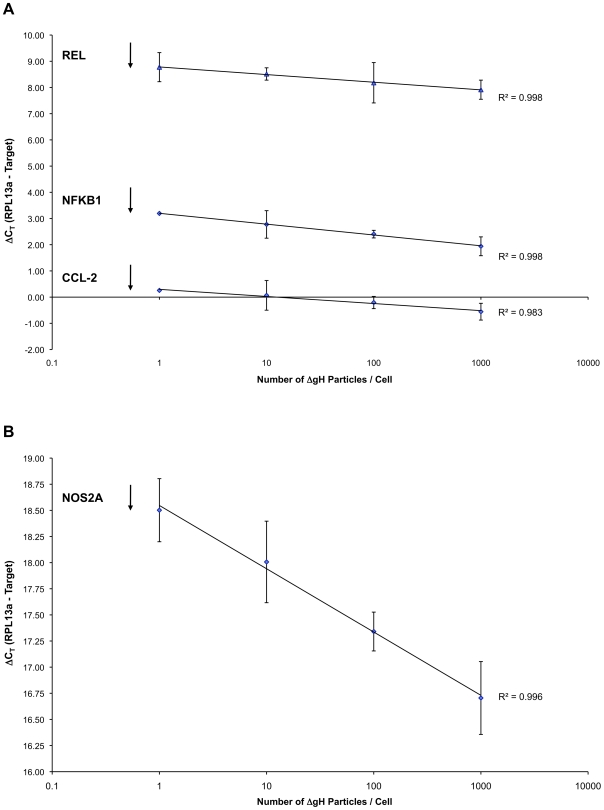
Effects of virion multiplicity on gene transcription. Low multiplicities of infection, which may represent physiological conditions, were sufficient to trigger an intracellular signalling response. (A) As the number of inoculated ΔgH particles increases, the number of cycles taken to reach the cycle threshold decreases for the NF-KB-responsive genes *nfkbi*, *ccl2* and *rel*, indicating an increased abundance of transcript. (B) A similar dose response is seen for the JAK/STAT target *nos2a*. Error bars are the +/− standard error across triplicate biological replicates.

The NF-κB responsive genes NFKB1, REL and CCL2, in addition to the JAK/STAT target NOS2A, were used as they had previously been shown to be differentially expressed after inoculation with 1000 particles/cell of ΔgH HSV-1. Recalling that the more abundant a transcript the fewer number of cycles it takes to cross a threshold set in the exponential phase of PCR product accumulation (the C_T_ value), Figure 2 plots the ΔC_T_ value on the y-axis, (i.e. the C_T_ value for the gene of interest after normalisation against the RPL13) against the multiplicity of infection on the x-axis. As the multiplicity of ΔgH increases the C_T_ value decreases, indicating an increase in the abundance of transcripts for all three NF-κB targets (Figure 2(a)). A logarithmic regression analysis across three logs of multiplicity show a high correlation between the four C_T_ values for each transcript with r^2^>0.98. Similarly for the JAK/STAT responsive target NOS2A there are almost two cycles of a difference between the level of transcripts after infection with 1 particle/cell compared to 1000 particles/cell (Figure 2(b)). This larger response than those for NF-κB responsive genes is indicative of the different level of induction shown with previous real-time PCR analysis of these transcripts (Table 2).

Arrows in Figure 2 indicate the C_T_ value for each of the four targets examined. Inoculation with 1 particle cell gave no detectable increase in transcript abundance but an increase was observed using 10 particles per cell and the dose response curves strongly suggest that lower doses have an effect. Interpreting these data in the context of infection is not straightforward as particle∶infectivity ratios for herpes simplex virus are, at best, 20 or more. On this basis a single infectious unit per cell is clearly sufficient to increase transcript abundance, indicating that signalling occurs at ‘physiological’ multiplicities of infection.

## Discussion

Evidence from previous studies using soluble HSV-1 glycoprotein and UV-inactivated virus suggested that binding of HSV-1 virions to the cell surface might be sufficient to stimulate intracellular signalling pathways. We undertook preliminary microarray studies with entry-defective HSV-1 virions and identified a number of pathways that were stimulated at early time points during infection.

Microarray experiments are inevitably vulnerable to false positives and negatives due to non-specific binding of labelled cDNA probes, necessitating a more robust follow-up with highly sensitive methods. Due to such confounders, these data were used exclusively as a guide to select, for real-time PCR, the transcriptional targets of intracellular signalling pathways stimulated after inoculation with entry-defective HSV-1.

**Figure 3:**
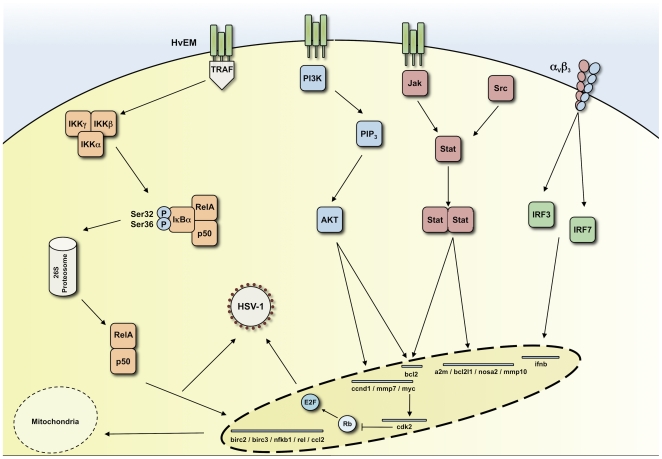
Model for glycoprotein-receptor interactions in the induction of intracellular signalling pathways by HSV-1. Glycoprotein D acts as the main signalling molecule on the surface of the HSV-1 envelope. gH interacts with α_v_β_3_ integrins to potentially trigger the production of IFN-β, which is known to involve IRF-3 and 7 [48]. Binding by gD to HvEM may lead to the activation of TRAF molecules, which in turn stimulate the NF-κB signaling cascade. This pathway up-regulates a number of cellular genes in addition to augmenting early viral gene expression. NF-κB-responsive genes, *birc2 and birc3*, have an anti-apoptotic role, but paradoxically, inflammatory mediators such as *ccl2* are also up-regulated. gD-induced signalling of the Jak/Stat and Jak/Src pathways also results in the differential expression of genes associated with anti-apoptosis and inflammation. The up-regulation of c-Myc could lead to a corresponding increase in *cdk2*, which has a role in promoting DNA replication and gene transcription during infection. It should be noted that most signalling cascades have been elucidated in non-fibroblast cells lines, so the role of specific kinases may vary in HFFs.

Gene targets associated with the NF-κB, JAK/STAT/Src, and PI3K/Akt pathways were shown to be differentially expressed after inoculation with glycoprotein-deficient virions, with the majority of signalling events being associated with the presence of gD on the envelope. Nineteen other signalling pathways present on the preliminary microarray experiments, and confirmed by real-time PCR, were not stimulated (data not shown). These results are compatible with our model, shown in Fig. 3.

Real-time PCR confirmed that changes in transcription associated with the NF-κB, JAK/STAT, JAK/Src and PI3K pathways were modulated as a result of virion binding, all of which required gD on the envelope surface To demonstrate that signalling occurred at physiologically relevant multiplicities of infection, HFFs were inoculated with either 1000, 100, 10 or 1 particles per cell of entry-defective HSV-1. Changes in gene transcription occurred in a dose-dependent manner and were detectable at 10 virus particles per cell. Given the particle∶infectivity ratios usually quoted for HSV1, we argue that this corresponds to physiological conditions (i.e. less than one infectious unit per cell), but it is impossible to know the circumstances that pertain *in vivo* when a single cell becomes infected. What seems almost certain is that an infected cell will normally present an uninfected neighbouring cell with 10 or more progeny particles.

HSV-1, as well as beta- and gammaherpesviruses, are capable of stimulating the NF-κB pathway in a bi-phasic manner, with our data supporting that an early, transient induction is reliant on virions expressing gD [8] [32]–[33]. Suppression of NF-κB activity is via negative feedback up-regulation of the inhibitor IκBα (*nfkbia*), which was also stimulated by the binding of entry-defective HSV-1 virions. The triggering of early NF-κB transcriptional activity was most likely through the coupling of gD on entry-defective virions to the TNF superfamily receptor HvEM [1]. In doing so, not only does the initial activation of this pathway allow for the subsequent sequestration of the NF-κB p65 subunit to the ICP0 promoter, but is crucial for immediate-early gene transcription and subsequent HSV-1 replication [8].

Intracellular signalling induced by soluble gD can protect against Fas-mediated apoptosis with inhibition of NF-κB signalling leading to a loss of this protection [6]. Infection with UV-inactivated virions also led to an increase in the expression of the anti-apoptotic protein c-IAP2 (*birc3*), which we have demonstrated to be up-regulated after inoculation with entry-defective virions containing gD. Additional studies have supported the anti-apoptotic role for NF-κB during HSV-1 infection yet there are conflicting data that demonstrate possible pro-apoptotic activity [34] [35]. This inconsistency may be due to differing cell types used in those studies. Primary human foreskin fibroblasts have been shown to be resistant to apoptosis after infection with recombinant HSV-1 that is unable to express ICP4 or ICP27 whereas infection with either virus has been shown to cause apoptosis in transformed cell lines [36].

Aspects of the HSV-1 life cycle, such as stimulating the progression of the cell cycle in the absence of serum, may be sufficient to induce a stress response and trigger apoptosis. Both *bcl2* (Bcl-2) and *bcl2l1* (Bcl-xl) belong to the Bcl-2 family of apoptosis regulators that provide cellular protection from a range of harmful stimuli such as cytokine deprivation, UV- and γ-irradiation [37]. Bcl-2 and Bcl-xl are found in the outer mitochondrial membrane and are thought to suppress apoptosis by blocking mitochondrial outer-membrane permeabilisation through the sequestration of pro-apoptotic Bcl2 family members [38]. Given the up-regulation of four anti-apoptotic genes, *birc2*, *birc3*, *bcl2* and *bcl2l1*, through the activation of multiple signalling pathways by entry-defective HSV-1, this establishes a role for gD binding in shifting the intracellular environment towards a more anti- apoptotic stance.

It is less apparent as to the biological relevance of an innate immune response stimulated through HSV-1 binding. It may be that there is a “cost” associated with altering the intracellular environment, which leads to the differential expression of cytokines, such as *ccl2*, that are under similar transcriptional regulation as those host factors that are favourable for virus replication.

Signalling by secreted Type I IFNs occurs through the Jak/Stat pathway results in the expression of various ISGs; a response that is also triggered by virus entry [39]. Nevertheless, productive infection with HSV-1 can down-regulate the triggered ISG response, allowing viral replication to continue unhindered [40]. Despite our evidence that gD binding by entry-defective virions can induce IFN-α mRNA expression, independent of gD glycosylation status, these data also fit with published observations that binding by HSV-1 is insufficient to cause the up-regulation of interferon-stimulated genes [30]. The up-regulation of IFN-β, albeit it through a different mechanism, is suggestive of a previously unidentified role for gH in eliciting a change in host gene expression. An entry-defective HSV-1 mutant lacking gB that also contains an RGE rather than the integrin binding RGD motif of gH has been constructed and future studies may further elicit the role of gH in interferon stimulation.

The methodology used here required the serum-starvation of primary human fibroblasts for five days. In the absence of serum, primary fibroblasts rapidly enter a quiescent state. As a DNA virus that requires host nuclear factors to replicate its genome, it is therefore not surprising that HSV-1 would stimulate cells from a G0 state into one that would favour DNA replication and possibly promote the transcription of viral genes.

Quiescent cells *in vitro* have very low levels expression of the transcription factor c-Myc. Its up-regulation is rapidly induced after mitogenic stimulation or the introduction of serum and increased expression of c-Myc is consistent with the advancement of cellular proliferation [41]. Control of *myc* transcription can be influenced by a number of pathways, including PI3K/Akt signalling, which was shown here to occur as a result of binding by gD. A central role for c-Myc in promoting cell-cycle progression is evident from the genes that it can up-regulate such as eIF2 [42]. Progression of the cell cycle depends on the additional activity of cyclin-dependent kinases. By interacting with the promoters for genes encoding cyclins and cyclin-dependent kinases, c-Myc can influence the advancement on the cell cycle into the G1/S phase [43].

Cyclin-dependent kinase 2 (CDK2) is one such downstream target of c-Myc activity, as well as the Androgen pathway, highlighting the signalling cross-talk that may occur [44]. CDK2 is involved in the progression of the cell cycle from G1 through to S phase. Transient activation of CDK2 was shown to occur early in HSV-2 infection at two hours post-infection, and is crucial in early HSV-1 infection [45] [46]. Kinase action by the cyclin A/CDK2 complex liberates the bound transcription factor E2F from Rb, a transcription factor that has previously been shown to be active during HSV-1 infection [47].

Epithelial cells at the initial site of HSV-1 infection *in vivo* are likely to be in a resting state, necessitating the virus to evolve a pre-entry signalling mechanism by which to stimulate the cell to provide host factors that are necessary for viral replication. We have demonstrated that signalling induced by HSV-1 glycoproteins, primarily gD, has the potential to: activate cellular transcription factors that augment viral gene transcription, differentially express a number of cellular genes so as to condition the cell for optimal replication or, alternatively, signal transduction may occur as a secondary effect to the appropriation of cellular receptors to achieve viral entry.

## Supporting Information

Table S1 Normalised datasets for genes differentially expressed on a signalling-specific microarray after infection with entry-defective HSV-1.
(0.14 MB XLS)

Table S2 Primers used in real-time PCR to determine the relative abundance of corresponding mRNA transcripts.
(0.28 MB DOC)

Figure S1 Characteristics of virions treated with PNGase.
(0.76 MB PDF)
