# Supplementary material for: Binding of Herpes Simplex Virus Type-1 Virions Leads to the Induction of Intracellular Signalling in the Absence of Virus Entry
Source: PLoS One. 2010 Mar 5;5(3):e9560. doi: 10.1371/journal.pone.0009560 (PMC2832691; doi:10.1371/journal.pone.0009560)
Supplement: Table S2 — Primers used in real-time PCR to determine the relative abundance of corresponding mRNA transcripts. (0.28 MB DOC) [file pone.0009560.s002.doc]

**Table S2.** Primers used in real-time PCR to determine the relative abundance of corresponding mRNA transcripts

| **Gene** | **Accession No.** | **Forward** | **Reverse** |
| --- | --- | --- | --- |
| **A2M** | NM_000014 | GAGCTTTGTCCACCTTGAGC | GCTTCACAAGCAGTCCATGA |
| **BCL2** | NM_000633 | GGATGCCTTTGTGGAACTGT | AGCCTGCAGCTTTGTTTCAT |
| **BCL2L1** | NM_138578 | TCTGGTCCCTTGCAGTAGT | CAGGGAGGCTAAGGGGTAAG |
| **BIRC1** | NM_022892 | GGTGAGGAATTCGAGACCAG | TGAGACAGACAGGGTCTTGC |
| **BIRC2** | NM_001166 | CCAGGTCCCTCGTATCAAAA | AAACCAGCACGAGCAAGACT |
| **BIRC3** | NM_001165 | CCAAGTGGTTTCCAAGGTGT | TTTCATCTCCTGGGCTGTCT |
| **BMP2** | NM_001200 | CCCACTTGGAGGAGAAACAA | GCTGTTTGTGTTTGGCTTGA |
| **BMP4** | NM_001202 | TGAGCCTTTCCAGCAAGTTT | CTTCCCCGTCTCAGGTATCA |
| **CCL2** | NM_002982 | CCCAGTGTCATCCTGGTCTT | GAGACTGTTCTGTGCGTCCA |
| **CCND1** | NM_053056 | AACTACCTGGACCGCTTCCT | CCACTTGAGCTTGTTCACCA |
| **CDK2** | NM_001798 | ATGTGACCAAGCCAGTACCC | TCACCCCTGTATTCCCAGAG |
| **CDKN1A** | NM_000389 | ACTTCCTCCTCCCCACTTGT | AGGTGAGGGGACTCCAAAGT |
| **CDKN2A** | NM_058195 | ATATGCCTTCCCCCACTACC | CCCCTGAGCTTCCCTAGTTC |
| **CDKN2B** | NM_004936 | GACCGGGAATAACCTTCCAT | CACCAGGTCCAGTCAAGGAT |
| **CXCL9** | NM_002416 | CCACCGAGATCCTTATCGAA | CTAACCGACTTGGCTGCTTC |
| **EGFR** | NM_201283 | GGTGCAGGAGAGGAGAACTG | GGTACGTGGTGGGGTTGTAG |
| **EGR1** | NM_001964 | TGACCGCAGAGTCTTTTCCT | TGGGTTGGTCATGCTCACTA |
| **FN1** | NM_054034 | ATCACCCTCACCAACCTCAC | CACTGTGACAGCAGGAGCAT |
| **FOS** | NM_005252 | CCAACCTGCTGAAGGAGAAG | GCTGCTGATGCTCTTGACAG |
| **GADD45A** | NM_001924 | AAGGGGCTGAGTGAGTTCAA | TTTTCCTTCCTGCATGGTTC |
| **HOXA1** | NM_005522 | TACCCCTCGGACCATAGGAT | GAGTAGGACACCCCCAGGTT |
| **ICAM1** | NM_000201 | GGCTGGAGCTGTTTGAGAAC | ACTGTGGGGTTCAACCTCTG |
| **IFNA1** | NM_024013 | GCAAGCCCAGAAGTATCTGC | ACTGGTTGCCATCAAACTCC |
| **IFNB1** | NM_002176 | TGGGAGGATTCTGCATTACC | CAGCATCTGCTGGTTGAAGA |
| **IGFBP3** | NM_000598 | CAGAGACTCGAGCACAGCAC | GATGACCGGGGTTTAAAGGT |
| **IL29** | NP_742152 | GAAGCAGTTGCGATTTAGCC | GAAGCTCGCTAGCTCCTGTG |
| **IRF1** | NM_002198 | CCTCTGCCTTCTTCCCTCTT | CTCTAGCCAGGGTCTCATGC |
| **IRF3** | NM_001571 | GAGGTGACAGCCTTCTACCG | TGCCTCACGTAGCTCATCAC |
| **IRF7** | NM_001572 | TACCATCTACCTGGGCTTCG | GCTCCATAAGGAAGCACTCG |
| **ISGF3G** | NM_006084 | TTGAGAGGGGCATCCTAGTG | GCCCTGAAAGTACCTGACCA |
| **ISG54** | NM_001547 | GCACCTCAAAGGGCAAAAC | GCTTCAGAGCCAGGAGGACT |
| **JUN** | NM_002228 | CCCCAAGATCCTGAAACAGA | CCGTTGCTGGACTGGATTAT |
| **JUNB** | NM_002229 | TCTCTCAAGCTCGCCTCTTC | ACGTGGTTCATCTTGTGCAG |
| **LEP** | NM_000230 | GGCTTTGGCCCTATCTTTTC | CCAAACCGGTGACTTTCTGT |
| **LTA** | NM_000595 | CACCGGAGCTTTCAAAGAAG | TGCTCTTCCTCTGTGTGTGG |
| **MMP7** | NM_002423 | GAGTGCCAGATGTTGCAGAA | AAATGCAGGGGGATCTCTTT |
| **MMP10** | NM_002425 | AAGTTCCTTGGGTTGGAGGT | TCAATGGCAGAATCAACAGC |
| **MYC** | NM_002467 | TTCGGGTAGTGGAAAACCAG | CAGCAGCTCGAATTTCTTCC |
| **NFKB1** | NM_003998 | CCTGGATGACTCTTGGGAAA | TCAGCCAGCTGTTTCATGTC |
| **NFKBIA** | NM_020529 | GCAAAATCCTGACCTGGTGT | GCTCGTCCTCTGTGAACTCC |
| **NFKBIB** | NM_002503 | GCTGGAGGCTGAAAACTACG | CTCAGGAGAAGCTCCAGCAC |
| **NOS2A** | NM_000625 | GCCCAGGTTCTACTCCATCA | ACATCCCCGCAAACATAGAG |
| **ODC1** | NM_002539 | CCCAGCGTTGGACAAATACT | TCCATAGACGCCATCATTCA |
| **PECAM1** | NM_000442 | AGACAACCCCACTGAAGACG | TGGGACCAGATCCTTCATTC |
| **RBP1** | NM_002899 | AGTGGATCGAGGGTGATGAG | GGACATTTTTGCCTCATGCT |
| **REL** | NM_002908 | CGGTTCAATTGGAGAAGGAA | CCATTGAGGCATGATGTGAC |
| **RPL13A** | NM_012423 | CCTGGTCTGAGCCCAATAAA | ACTTCGGGAGGCAGTGACTA |
| **TMEPAI** | NM_020182 | TAAGCTCTCGGCTTCTCTGC | GCCTTCTCTGAACCAGGATG |
| **WISP2** | NM_003881 | CTCCCTGCCTACACACACAG | AAACTCCAGAAAAGGCAGCA |
