# Supplementary material for: Binding of Herpes Simplex Virus Type-1 Virions Leads to the Induction of Intracellular Signalling in the Absence of Virus Entry
Source: PLoS One. 2010 Mar 5;5(3):e9560. doi: 10.1371/journal.pone.0009560 (PMC2832691; doi:10.1371/journal.pone.0009560)

Figure S1. Characteristics of virions treated with PNGase

a) PNGase treatment of wild-type HSV-1 virions has no notable effect on infectivity when compared to untreated preparations. All samples had titres of the order of 10^9 pfu / ml on HFF cells. Titres include the standard error from duplicate assays. b) ΔgH-negative virions were incubated with PNGase for 18 hours at 37°C. The loss of N-linked sugars on envelope glycoproteins resulted in an electrophoretic mobility shift of gD (Lane 2) when compared to untreated virus particles (Lane 1). There was no change in VP16 mobility.

A

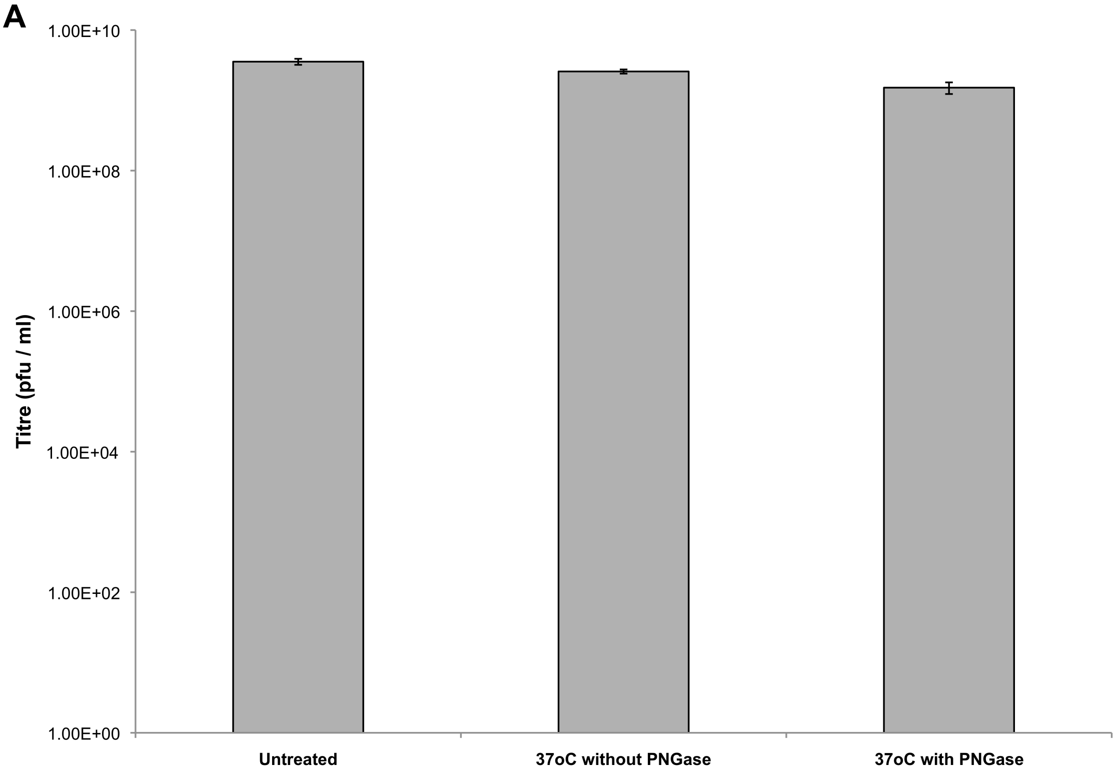

B

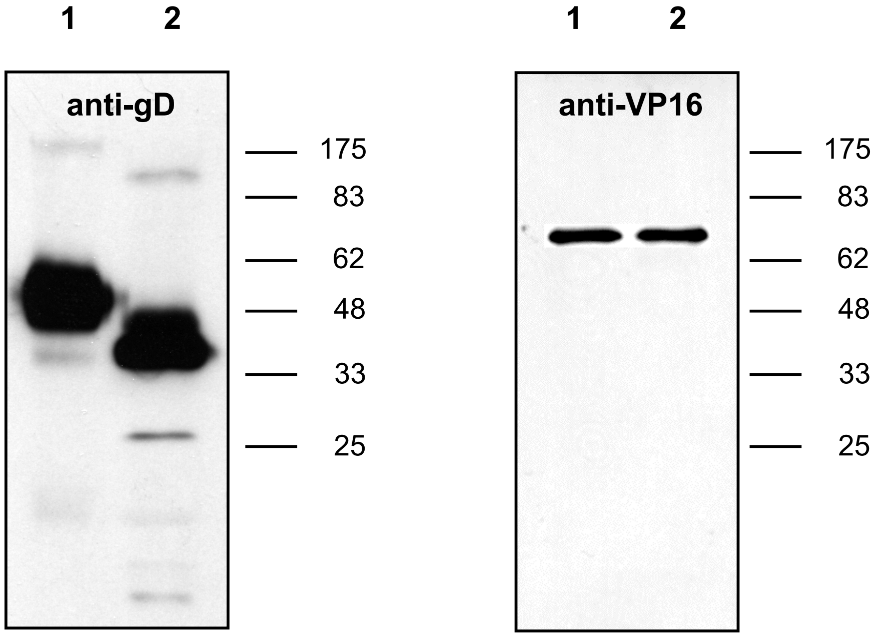

Supplement: Figure S1 — Characteristics of virions treated with PNGase. (0.76 MB PDF) [file pone.0009560.s003.pdf]
